# COVID-19 during malaria transmission season in Africa and why we should be prepared: An example from Senegal

**DOI:** 10.4102/ajlm.v9i1.1332

**Published:** 2020-11-18

**Authors:** Khadim Diongue, Mamadou A. Diallo

**Affiliations:** 1 Parasitology-Mycology Service, Faculty of Medicine, Pharmacy and Odontology, Cheikh Anta Diop University, Dakar, Senegal; 2 Laboratory of Parasitology and Mycology, Aristide Le Dantec University Hospital, Dakar, Senegal

## Introduction

Coronavirus disease 2019 (COVID-19) is not just a global health crisis; it is also a socio-economic crisis^1^ with a huge number of associated deaths.^2^ On 30 June 2020, the World Health Organization reported 10 185 374 cases and 503 862 deaths related to COVID-19 globally, of which 297 290 cases and 6010 deaths were recorded in Africa.^1^ In Senegal, malaria transmission is seasonal with high vector populations observed during the rainy season – from mid-June to October. The heaviest rainfalls are observed in the months of July, August and September. On 30 March 2020, as effort to combat the spread of COVID-19 in Senegal, a state of emergency was declared banning religious gatherings, closing schools and universities and imposing a curfew between 22:00 and 06:00. As of 30 June 2020, the day the state of emergency was lifted, the authorities had reported 6698 confirmed COVID-19 cases and 108 COVID-19 related deaths^1,3,4^ thus the country was confronted with managing the expected rainy-season malaria epidemic and the on-going COVID-19 pandemic. Hence, this article aims to draw the attention of authorities to this current situation.

## An important epidemiological reminder in this current context

Before and after the ease of lockdown measures on 04 June 2020, Senegal had been experiencing constantly increasing COVID-19 cases, as evidenced by a 22% increase in cases during the month of June 2020. There were 1838 COVID-19 cases on 01 June 2020, which had reached 2249 cases by 30 June 2020. However, the most detrimental event during the COVID-19 pandemic is that of the general population deserting the health facilities that are hosting infected COVID-19 individuals for fear of being tested or infected. Since the decentralisation and proliferation of COVID-19 care centres around the country, COVID-19 hosting centres are being avoided by the populace; worse still, patients have now deserted the isolation centres. Senegalese people avoid going to places where any test that could suggest COVID-19 is done, even a body temperature measurement. However, experience has shown that in an outbreak such as the COVID-19 pandemic, it is essential to not ignore other diseases such as malaria. The recent West Africa Ebola epidemic revealed the deleterious impact of increased health service demand on an already fragile health system, as well as on the control of other diseases. Consequently, the Ebola outbreak led to a substantial increase in morbidity and mortality of other diseases, including malaria.^5^ The World Health Organization has warned of a twofold devastation by the COVID-19 pandemic: the first is its direct effect on health – COVID-19 morbidity and mortality – and the second is the related increase in morbidity and mortality of other diseases due to lack of or diversion of adequate health response for other diseases.^5^ For example, during the Ebola epidemic year in Guinea, health facilities recorded a lower malaria patients’ visitation than was expected – an estimated deficit of 74 000 visitations. Conversely and sadly, malaria caused 1067 deaths in 2014 – an Ebola year – compared to 108 deaths reported in 2013 – a non-Ebola year.^6^ More worryingly, in Liberia, Sierra Leone and Guinea, about 7000 other deaths related to malaria were recorded among children under age 5 years, which were attributed to the 2014–2016 Ebola outbreak.^6^

## What we know

Malaria illness presents some similar symptoms to the COVID-19 illness: fever, respiratory distress, fatigue and an acute onset headache.^5,7^ The major sign between both illnesses is fever. Thus, a malaria case or a COVID-19 case may each be confused for the other, if symptoms alone are used to define a case during this current pandemic. Also, since the beginning of the pandemic in Senegal, fever is the major symptom of infected individuals^8^; febrile individuals are isolated before samples are taken. The sampling team is deployed after contact with the Senegalese medical emergency assistance service; however, the emergency assistance centre has been overwhelmed (more than 726 000 emergency calls between 02 March 2020 and 29 June 2020^9^), which could be the reason why, unfortunately, sampling teams can sometimes take more than 24 h to intervene. This delays the differential diagnosis of malaria and similar illnesses, as well as increases the risk of development of severe malaria, if malaria diagnosis is correct but delayed. Therefore, clinicians should go back to the good old method of ‘any case of fever must be considered as malaria until proven otherwise’. Confirming either malaria or severe acute respiratory syndrome coronavirus 2 (SARS-CoV-2) infection with a diagnostic test does not rule out the possibility of the other in a patient. Indeed, both false-positive and false-negative results have been reported worldwide with rapid diagnostic tests.^10^ Likewise, approximately 50% of infected cases are likely missed by current screening approaches for COVID-19, even in countries with good healthcare systems and available diagnostic capacities. As COVID-19 can be transmitted even by asymptomatic people, and personal social distancing cannot be effective when performing a malaria test, such as a rapid diagnostic test,^11^ other infection prevention and control measures must be carefully put in place.^7^ These measures include but are not limited to: proper donning and doffing of personnel protection equipment, especially face masks; provision and use of soap and water for handwashing; provision and use of alcohol-based hand sanitiser and the practice of good respiratory hygiene (i.e. covering of the mouth and nose with a disposable tissue when sneezing or coughing or sneezing into the crease of the elbow).^7^ Thus, health providers should also be more careful.

## What we should do

In Senegal, according to the last national malaria control programme report published in 2018, malaria caused 395 706 cases with 284 deaths including 95 deaths (33.45%) among children younger than age 5 years.^12^ However, the malaria burden was more than 50% lower between 2009 and 2015, permitting the country to achieve the objectives of Roll Back Malaria in 2015.^3^ Therefore, to not lose this positive momentum in the fight against malaria, the Senegalese national malaria control programme should scrupulously follow the recommendations of the World Health Organization, which include tailoring malaria interventions in the COVID-19 response^5^ with particular regard to the following points: case management, chemoprevention (intermittent preventive treatment in pregnancy and seasonal malaria chemoprevention); extraordinary interventions, including presumptive treatment of fever and mass drug administration, as well as the assurance of continued access to and use of recommended insecticide-treated mosquito nets; and implementation of core vector-control activities to the greatest extent possible (current and planned insecticide-treated nets campaigns should go ahead, if at all possible). Malaria prevention and treatment is even more important during the COVID-19 pandemic than under normal circumstances.

Rapid diagnosis plays an important role in the outcome of both malaria and COVID-19; thus, in addition to these recommendations, quick, easy, reliable and cheap diagnostics requiring minimal invasion must be performed, especially in cases of likely overlap with malaria and COVID-19. This is the time for malaria-endemic countries challenged with the COVID-19 pandemic to apply modern techniques including point-of-care tests such as the Loop-Mediated Isothermal Amplification assay for rapid detection of both *Plasmodium* and SARS-CoV-2.^13,14^ Meanwhile, health authorities should also centralise the management of COVID-19 infected individuals so that populations suffering from other diseases (diabetes, high blood pressure, cardiovascular disorders, arthritis, etc.) could return to health facilities without fear of being infected. However, number of tests as well as the test centers for COVID-19 detection should be multiplied in order to attend benefits of early case identification.
